# Midterm Results of a New Rotating Hinge Knee Implant: A 5-Year Follow-Up

**DOI:** 10.1155/2017/7532745

**Published:** 2017-12-10

**Authors:** Christoph Böhler, Paul Kolbitsch, Reinhard Schuh, Richard Lass, Bernd Kubista, Alexander Giurea

**Affiliations:** Department of Orthopaedic Surgery, Medical University of Vienna, Waehringer Guertel 18-20, 1090 Vienna, Austria

## Abstract

**Background:**

In the current study, we investigated midterm results of a new rotating hinge total knee arthroplasty (EnduRo prosthesis), which uses a new bearing material (CFR PEEK).

**Methods:**

We prospectively analysed data of 50 patients with a minimum follow-up of 5 years. In 24 (48%) patients, a primary implantation was performed and 26 (52%) were revision cases. Clinical and radiographic examinations were performed preoperatively as well as postoperatively after 3 and 12 months and annually thereafter. The Knee Society Score (KSS), WOMAC, Oxford Knee Score (OKS), and range of motion (ROM) were used for clinical assessment.

**Results:**

KSS, WOMAC, OKS, and ROM significantly improved between the preoperative and the follow-up investigations. The overall survival rate with revision for any reason as an endpoint was 77.9% after five years. The number of complications was significantly higher in the revision group (*p* = 0.003).

**Conclusion:**

The EnduRo prosthesis provides highly satisfying clinical and functional results in severe primary as well as in revision cases. Implant-associated complications were rare. However, in cases of revision surgery, the risk for complications was considerably high, mostly related to previous joint infections and poor soft tissue quality.

## 1. Introduction

The number of primary and revision total knee arthroplasties (TKA) is steadily rising. In the Swedish knee arthroplasty register, the frequency of revisions has increased to 13% [1] and in the United States prosthetic revisions account for approximately 8% of all total knee replacements [2]. In revision TKA, the choice of an adequate constraint degree represents one of the crucial factors for a successful outcome [3]. In cases with serious bone loss and ligament instability, more constrained designs, like rotating hinge prosthesis, are necessitated [4, 5]. Beside revision arthroplasty, rotating hinge prostheses are also used in primary cases with excessive varus/valgus deformities (>20°), severe rheumatoid arthritis, collateral ligament insufficiencies, or bony destruction of the tibial plateau or the femoral condyles [6].

Historically, hinged knee prostheses were the first implants in TKA [7]. First-generation hinge prostheses had only one plane of motion. The highly restricted biomechanics and the suboptimal designs caused early loosening, osteolysis, and excessive wear [8–10]. The poor results lead to several developments including a rotational axis that additionally allowed 20° of internal and external rotation. However, the outcomes of these second-generation implants remained disappointing [11, 12]. Further enhancements, including a new design of the trochlear groove to improve patella tracking and patella kinematics, advancements in the stem design that facilitate osteointegration, improvement of biomaterials, and addition of second rotational axis to decrease torsional stresses on the bone implant interface, were introduced [8, 13–15].

Despite these improvements, the results and indications are still discussed contradictorily. Certain authors report high complication rates and low survivorship and therefore consider rotating hinge implants to be only useful in salvage procedures after several failed revisions [16]. However, other studies reported encouraging outcomes and recommend a more liberal usage [6, 17, 18]. Many previous reports on modern rotating hinge implants are done retrospectively and, due to the usage of different types of prosthesis, are difficult to compare [19].

After early result evaluation [20], in the current study we investigated midterm clinical, functional, and radiological outcomes, complications, and implant survival of a new third-generation rotating hinge prosthesis (EnduRo prosthesis; Aesculap AG, Tuttlingen, Germany), which uses a new bearing material (carbon-fiber reinforced poly-ether-ether-ketone, CFR PEEK) in complex primary and revision patients. Furthermore we aimed to compare outcomes and complications between primary and revision implantation and analysed risk factors for complications.

## 2. Material and Methods

### 2.1. Patients

In this single centre study, we prospectively collected data from 59 patients who underwent surgery between 2008 and 2012. In all patients, the EnduRo rotating hinge prosthesis was implanted. The regional institutional review board gave ethical approval for this study and all patients signed an informed consent before study inclusion. Five patients died from unrelated causes and four patients were unable to participate because of severe comorbidities, leaving 50 patients with a minimum follow-up of 5 years for final analysis. In 24 (48%) patients, a primary implantation was performed and 26 (52%) were revision cases. Indications for primary implantation included varus or valgus osteoarthritis of more than 20° deformity and severe rheumatoid arthritis in combination with extensive ligamentous instability and/or bony destruction. Indications for revision TKA included aseptic and septic loosening with substantial bone loss as well as serious instability after primary TKA, including flexion and extension gap mismatch. Differentiation between aseptic and septic loosening was based on histological examination and microbiological diagnostics as well as clinical and radiological examination combined with blood examinations, including C-reactive protein (CRP) and white blood cell (WBC) counts. In case of PJI, patients were treated by two-stage revision. The Charlson Comorbidity Index was applied to summarize comorbidities [21].  Table 1 summarizes the demographic data and indications for implantation.

### 2.2. Implant Design

The EnduRo prosthesis is a new modular rotating hinge implant. The transmission of force travels from the femoral component to the tibial part via the polyethylene (PE) insert, whereas the axis is primarily not weight bearing and stabilizes the implant when higher frontal and sagittal forces occur. The contact area between the metal parts and the PE is considerably large, varying from 680 to 1050 mm^2^ during the entire range of motion (ROM). PE inserts are available in 10 to 24 mm thickness. The axes are embedded in bushings and flanges made of CRF-PEEK, a novel biomaterial introduced as bearing articulation in knee arthroplasty for the first time aiming to minimize wear [22, 23]. The prosthesis is designed for a ROM from 3° of hyperextension to 140° of flexion and allows a rotation of ±12°. Tibial and femoral (posterior distal and distal) wedges in different heights allow for augmentation. Furthermore the prosthesis provides an offset option for femoral and tibial stems. Cemented and cementless stem fixation are both possible, whereas the epiphyseal fixation of the implant has to always be cemented [20].

### 2.3. Surgical Technique

All surgeries were carried out by the senior author (AG). The same approach was used for each intervention, involving a straight midline incision combined with a medial parapatellar arthrotomy and a lateral patella luxation. A combination of intramedullary femoral and extramedullary tibial alignment guides as well as resection blocks for osteotomies was applied. Spacers were utilized to balance flexion and extension gaps. A tourniquet was activated after osteotomies to improve cement penetration and was released before wound closure. For implant fixation, a hybrid technique of cemented femoral and tibial epiphyseal fixation and uncemented stems was exercised. After pulsatile lavage Gentamycin-loaded (Palacos R + G, Heraeus, Hanau, Germany) cement in vacuum cementing technique was applied. The patella was routinely resurfaced; if necessary, a lateral release was performed to obtain a satisfying patella tracking. Usually two intra-articular suction drains were set and kept for two to five days. Mobilization under physiotherapeutic surveillance already started on the first postoperative day. Patients used crutches for 6 weeks with partial to full weight bearing where possible. They received a perioperative antibiotic prophylaxis with Cefazolin or Clindamycin, which was usually continued for 5 days. In case of two-stage revision due to PJI, antibiotic therapy was adjusted according to culture results in consultation with the department of infectious diseases. Routinely, antibiotics were applied for six weeks between stages and continued for another 6 weeks after replantation. Thromboembolic prophylaxis was given throughout six weeks, starting 12 hours after the intervention.

### 2.4. Follow-Up and Complications

Clinical and radiographic examinations were performed preoperatively as well as postoperatively after 3, 6, and 12 months and annually thereafter. ROM was measured in action using a long goniometer. The Knee Society Score (KSS), the functional KSS [24], the Western Ontario and McMaster Osteoarthritis Index (WOMAC) [25], and the Oxford Knee Score [26] were used for clinical assessment. Two different observers evaluated standard anteroposterior, lateral, and full leg radiographs. Radiographic analysis included assessment of alignment and signs of loosening (radiolucent lines, osteolysis, PE wear, and implant migration). Furthermore, we evaluated complications leading to revision surgery of the involved knee and differentiated between three different types of complications as previously described [20]: type 1, PJI; type 2, periprosthetic complications such as failure of the extensor mechanism, periprosthetic fractures, patella failure, and wound dehiscence; type 3, implant complications like wear, aseptic loosening, instability, and implant failure (including breakage of axis, bushings, and stem). This particular differentiation originates from the observation that the implant has no or little influence on type 1 (septic) and type 2 (periprosthetic complications) failures, whereas type 3 failures are specifically linked to the prosthesis and therefore provide information about implant survival [20].

### 2.5. Statistical Analysis

Descriptive statistics were used to display demographic data. Statistical analysis focused on evaluation of the clinical and functional outcome five years after implantation. We used the paired* t*-test to compare pre- and postoperative results. The implant-survival rate is described using the Kaplan Meier (KM) method and analysed with Cox regression. We evaluated the failure rates of each complication type (1 to 3). Furthermore, we compared clinical and functional results as well as implant survival of the primary and the revision surgery group. We used the KM method, the* t*-test, nonparametric tests, and the Chi-Square test to compare these two surgery groups. Odds ratios (ORs) and 95% confidence intervals (95% CIs) were employed to describe the influence of potential risk factors for revision, applying logistic regression as calculation method. Values ≤ 0.05 were considered as statistically significant. All statistical analysis was carried out with IBM SPSS Version 24.

## 3. Results

Overall, we found a significant improvement in clinical and functional parameters after surgery. The KSS improved from 24.9 (SD 19.6) preoperatively to 89.3 (SD 16.6) postoperatively (*p* < 0.001) and the functional KSS from 25.2 (SD 20.5) to 58 (SD 26.6) (*p* < 0.001), respectively. WOMAC scores decreased from 6.3 (SD 1.9) before surgery to 2.5 (1.9) after surgery (*p* < 0.001) and the Oxford Knee Score increased from 15.1 (SD 8.6) preoperatively to 31.4 (SD 10.1) (*p* < 0.001), respectively. The ROM improved from 71° (SD 34°) preoperatively to 115° (SD 14°) after implantation of the rotating hinge prosthesis. None of the patients required a postoperative closed manipulation. When comparing functional and clinical results of the primary and the revision group, we registered significantly higher KSS results only in the primary group (*p* = 0.043). Functional KSS, WOMAC score, Oxford Knee Score, and the ROM did not differ between the two groups.  Table 2 summarizes pre- and postoperative clinical and functional results. Although radiolucencies were seen in six cases, all remained under 1 mm in thickness and no progression could be detected.

In total, 11 (22%) patients had complications leading to revision surgery. Out of these, four (8%) were deep infections (type 1 complication). The affected patients were treated with two-stage revision with prosthesis removal, implantation of cement spacer, and replantation after 6 weeks. An EnduRo rotating hinge prosthesis was implanted in three cases; in a single case GMRS distal femur tumour prosthesis (Stryker, Warsaw, USA) was chosen. Type 2 (periprosthetic) complications were registered in three (6%) patients. One of these patients suffered a periprosthetic fracture, which was treated with locking plate osteosynthesis. In the other two patients, a rupture of the extensor mechanism occurred. These patients were treated with surgical extensor mechanism reconstruction and cast-immobilization for 6 weeks. Type 3 complications (prosthesis failure) were seen in four (8%) patients. Two showed an aseptic loosening of the femoral component and both were treated with one-stage revisions: in one patient again a hybrid technique was performed; for the other, the revision component was completely cemented. In the remaining two patients, a failure of the tibial rotational axis screw occurred. One breakage and one loosening of the screw were recorded. In both cases, the screw was exchanged. The number of complications was significantly higher in the revision group (*p* = 0.003). Of 26 patients in the revision group, ten (38.5%) developed a complication, whereas only one (4.2%) out of 24 patients in the primary group was affected. This patient suffered a periprosthetic fracture. Tables 1 and 2 compare the primary and the revision group.

**Figure 1:**
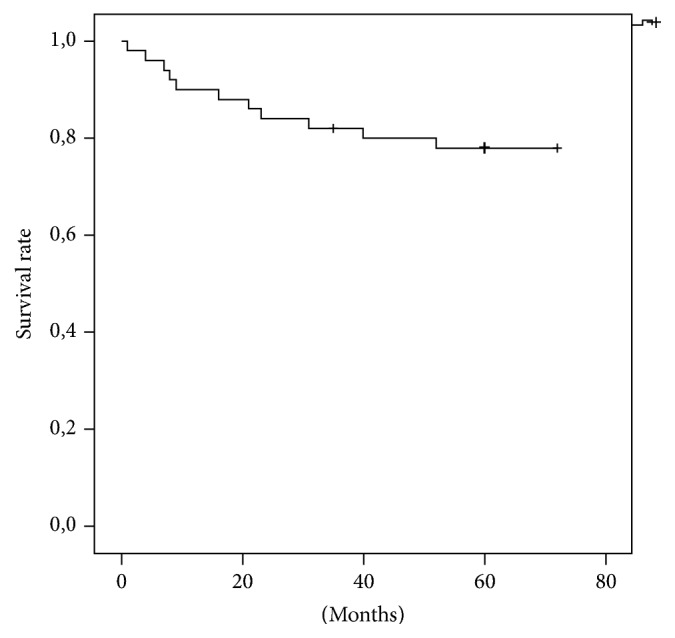
Revision-free survival (overall).

**Figure 2:**
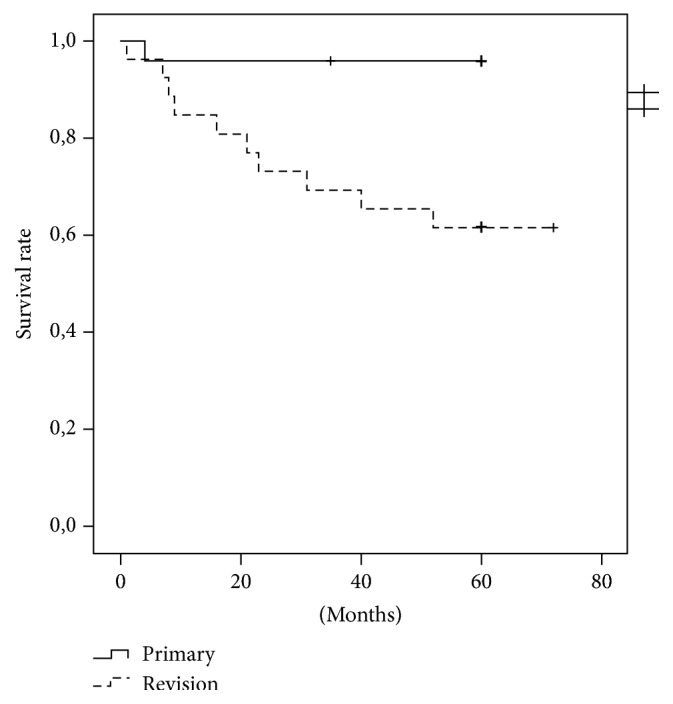
Comparison between primary and revision group.

**Figure 3:**
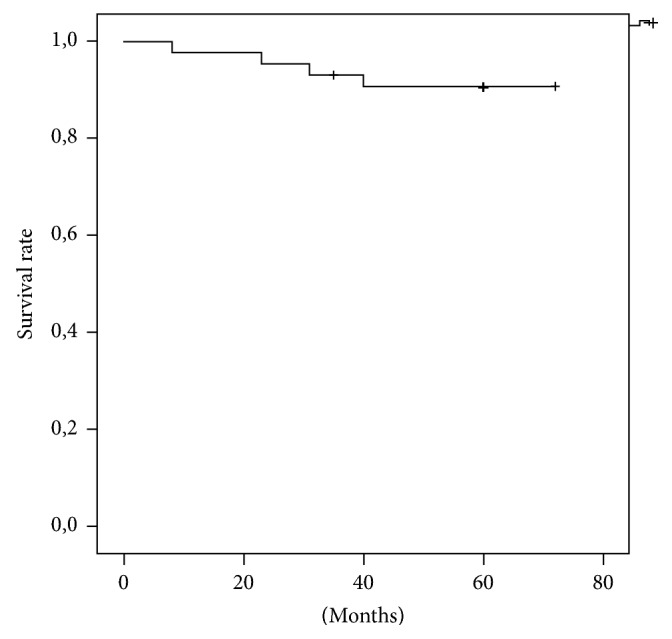
Implant survival (type 3 complication).

The overall survival rate with revision for any reason as an endpoint was 90% after one year and 77.9% after five years. Analogue to the complication rates, the survival was significantly higher in the primary group than in the revision group (*p* = 0.005). In the primary group, implant survival was 95.8% after one year and after five years, respectively. In the revision group, rates were 84.6% after one and 61.5% after five years, respectively. The implant-survival rate (type 3 complications) was 97.7% after one year and 90.6% after five years, respectively. See also Figures 1–3.

In addition, logistic regression was performed to evaluate independent risk factors for complications leading to revision surgery. In the model, revision surgery and age could be identified as influencing risk factors for complications. ORs were 14.38 (CI 95% 1.67–123.7) (*p* = 0.015) for revision surgery and 0.91 (CI 95% = 0.84–0.99) (*p* = 0.024) for age, respectively. Gender, CCI, and BMI did not show significant influences.

## 4. Discussion

The frequency of complex revision TKA is steadily increasing. Significant bone loss and ligament deficiency often require more constrained implants for reconstruction. Hinge prostheses have been attempted for decades, but prior designs still face complications in early aseptic loosening and implant failure due to the high stress placed on the bone-cement interface [27]. Despite several developments in prosthetic design, results as well as indications of rotating hinge devices are still discussed controversially [16–18]. In the current study, we prospectively evaluated the clinical and functional outcome as well as complication rates 5 years after implantation of a novel rotating hinge prosthesis, which was implanted for the first time in 2008. We found excellent clinical and functional results. Patients reported a tremendous improvement in daily living and quality of life. Overall complications leading to revision were seen in 22% of the cases after five years. The risk for complications was elevated after revision surgery and increased with age.

Our study has several limitations. First, the sample size was limited to 50. This has to be considered when interpreting our results; especially the influence of some risk factors for complications might have been underestimated. Second, the follow-up time was limited to five years. However, 50 cases evaluated prospectively over 5 years seem to be an acceptable number, in particular for a clinically unproven prosthesis design with a new bearing material. Third, the included patients showed distinct indications for a rotating hinge TKA. Potentially, the reason for revision surgery could impact the risk for complications, but the small sample size did not allow an evaluation of the influence.

After five years, patients had a significantly improved knee function. Overall mean KSS and functional KSS were 89 and 58, respectively, with a mean active knee flexion of 115°. The current KSS results are comparable with former studies on contemporary rotating hinge prosthesis, while the functional KSS results seem to be superior: Cottino et al. reported a mean KSS of 81 points and a mean functional KSS of 36 points in large cohort of primary and revision cases [28]. Pour et al. analysed 44 prostheses and KSS and functional KSS were 73.5 and 43, respectively [29]. Another study on rotating hinge prostheses in primary TKA found postoperative KSS of 73 and functional KSS of 47 after a mean follow-up of 15 years [19]. The better functional results might be related to the prosthetic design used in our cohort.

Overall 11 (22%) patients had a complication leading to revision surgery after five years. These results correspond with other reports, which described complication rates between 16% and 32% after 4- to 6-year follow-up [27–29]. Other published series found explicit lower revision-free survival rates after rotating hinge TKA with complications in 46% of the cases in a comparable follow-up period [30]. PJI was the most common complication and was seen in 8% of the cases. All of these patients had undergone revision TKA, because of previous septic loosening. In accordance with our study, deep infections accounted for most complications in previous reports, which described septic failure rates of 16% to 24% [19, 30].

Extensor mechanism complications are a frequent problem in revision TKA [31]. The incidence in our cohort of 4% seems to be comparable to or even a little lower than that in previous series that reported rates up to 10% of extensor mechanism complications [4]. One patient sustained a periprosthetic fracture; such frequency is in accordance with former reports, where one out of 42 patients suffered a periprosthetic fracture [4]. Periprosthetic fractures are more common after hinge prosthesis due to the higher constraint and the often aged patient collective suffering from additional risk factors like osteoporosis and rheumatoid arthritis [4].

Implant-associated complications were aseptic loosening and mechanical hinge failure (type 3 failure). In two patients (4%), we found a breakage of the tibial axis screw. Similar frequencies have been reported [15]. In contrast to early designs of constrained TKA, the presented aseptic loosening rate of 4% after 5 years was highly satisfying. This emphasizes the remarkable reduced shear stress on the bone implant interface by shifting the force transmission through the condylar area with additional rotating motion around the tibial axis. Considering implant-associated complications, we registered an implant-survival rate of 91% after 5 years.

None of the complications could be linked to the new CFR-PEEK bearing material. In vitro biotribologic studies have shown promising low rates of wear and debris for the EnduRo prosthesis [32]. We found no macroscopic noticeable signs of biological response. This clinical view is also supported by cell culture experiments showing that CFR-PEEKPAN wear particles had no cytotoxic effects [33]. A recent study described a complete different agglomeration behavior of UHMWPE and CFR-PEEK particles in human synovial tissue [22].

Patients undergoing revision surgery had a 14 times higher risk for a subsequent complication than patients after primary implantation. Besides the fact that revision surgeries are technically more demanding, many of the patients in the revision group had multiple prior operations (including revision surgery because of septic loosening) and therefore a very compromised bone and soft tissue quality. Among the remaining evaluated risk factors, only age had a significant influence on the complication rate. Though, as mentioned before, we have to admit that, due to the given sample size, the influence of some risk factors, especially the comorbidities, might have been underestimated. Further studies with larger cohorts are necessary to confirm our results.

## 5. Conclusion

In conclusion, the EnduRo rotating hinge prosthesis provides highly satisfying clinical and functional results in severe primary as well as in revision cases. We showed an overall survival rate of 78% after five years. Implant-associated complications were rare, underlining the design improvements of modern rotating hinge prostheses. However, in cases of revision surgery, the risk for complications was considerably high, mostly related to previous joint infections and poor soft tissue quality.

## Figures and Tables

**Table 1:** Patient characteristics and differences between primary and revision group. Except where indicated otherwise, values presented are mean (standard deviation).

	Total	Primary	Revision	*p* value
*n (%)*	50	24 (48%)	26 (52%)	
*Female n (%)*	40 (80%)	22 (91.7%)	18 (69.2%)	*p = 0.048*
*Age*	73 (1)	74.7 (2)	72 (1.7)	*p* = 0.177
*Weight (kg)*	82 (3)	79 (3)	85 (4)	*p* = 0.600
*Height (cm)*	165 (1)	164 (2)	167 (2)	*p* = 0.206
*BMI*	29.9 (0.9)	29.5 (1.1)	30.4 (1.4)	*p* = 0.749
*CCI*	6 (0)	7 (0)	6 (0)	*p* = 0.355
*RA n (%)*	3 (6%)	2 (8.3%)	1 (3.8%)	*p* = 0.504
*DM n (%)*	12 (24.5%)	6 (26.1%)	6 (23.1%)	*p* = 0.807
*Hospitalization (days)*	18	17 (1)	19 (1)	*p* = 0.558
*Indications n (%)*				
Primary	24 (48%)	—	—	
Septic loosening	10 (20%)	—	10 (38.5%)	
Aseptic loosening	11 (22%)	—	11 (42.3%)	
Instability	5 (10%)	—	5 (19.2%)	
*Complications (all)*	11 (22%)	1 (4.2%)	10 (38.5%)	*p = 0.003*
Type 1	4 (8%)	0	4 (15.4%)	
Type 2	3 (6%)	1 (4.2%)	2 (7.7%)	
Type 3	4 (8%)	0	4 (15.4%)	

**Table 2:** Preoperative and 5-year postoperative outcome parameters.

	Preoperative	5-year postoperative	*p* value
*WOMAC score (SD)*	6.26 (0.27)	2.45 (0.29)	*p < 0.001*
Primary	6.4 (0.37)	2.04 (0.38)	
Revision	6.13 (0.40)	2.84 (0.42)	
*Oxford Knee Score (SD)*	15 (1)	31 (1)	*p < 0.001*
Primary	15 (2)	34 (2)	
Revision	15 (2)	29 (2)	
*KSS clinical (SD)*	25 (3)	89 (2)	*p < 0.001*
Primary	15 (3)	92 (4)	
Revision	35 (4)	87 (3)	
*KSS function (SD)*	25 (3)	58 (4)	*p < 0.001*
Primary	23 (4)	60 (6)	
Revision	28 (4)	56 (5)	
*ROM (SD)*	72 (5)	115 (2)	*p < 0.001*
Primary	85 (4)	116 (3)	
Revision	60 (8)	114 (3)	
